# Utility of a Body Shape Index Parameter in Predicting Cardiovascular Disease Risks

**DOI:** 10.7759/cureus.23886

**Published:** 2022-04-06

**Authors:** Kawaiola C Aoki, Harvey N Mayrovitz

**Affiliations:** 1 College of Medicine, Nova Southeastern University Dr. Kiran C. Patel College of Osteopathic Medicine, Fort Lauderdale, USA; 2 Medical Education and Simulation, Nova Southeastern University Dr. Kiran C. Patel College of Allopathic Medicine, Davie, USA

**Keywords:** overweight, disease prevention, nhanes, absi, body shape, cardiovascular disease, central obesity, bmi, obesity

## Abstract

Background: Anthropometric indices are used as predictors of cardiovascular disease (CVD). The most used indices are body mass index (BMI) and waist circumference (WC); however, there are limitations regarding their validity to address different body shapes, fat and lean mass distribution. A body shape index (ABSI) has been proposed as an alternative parameter to reflect differences in body shape and potentially be more useful for predicting CVD. ABSI is calculated by ABSI = WC / (BMI^2/3 ^• Height^1/2^). The purpose of this cross-sectional study was to determine the utility of ABSI as a predictor or modifiable risk factor of CVD compared to other commonly used measures in clinical practice.

Methods: The sample population was from the baseline interview and health examination included in the National Health and Nutrition Examination Survey (NHANES) 2013-2014. Patients (n=5,924, 52% female) were aged 18-80 years (47.4 ± 18.4 years) who completed a series of questionnaires on a spectrum of health-related risks. After the interview, health technicians performed a standardized examination of the participants to collect data on weight, height, BMI, WC, and sagittal abdominal diameter (SAD). Statistical analysis was done using R Studio, version 0.99.903 (RStudio, Inc. Boston, MA). Using logistic regression, the correlation between each predictor (ABSI, BMI, WC, SAD) as a continuous variable, and CVD outcomes was evaluated with two models: a univariable model and a multivariable model. In a secondary analysis, ABSI was reclassified into categorical values based on quartiles of the NHANES dataset. Logistic regressions were again run for overall CVD and all CVD sub-categories, followed by chi-square tests for significance. For comparison, BMI categories of normal, overweight, obese, and severely obese were tested with overall CVD and all CVD subcategories as outcome measures, followed by chi-square tests for significance.

Results: Approximately 10% of the sample population had at least one prior manifestation of CVD, the most common being myocardial infarction (MI) (4.0%). ABSI showed little correlation with weight, BMI, WC, and SAD (r<0.3), while BMI had a strong correlation with weight, BMI, WC, and SAD (r ≈ 0.9). In univariable logistic regression, ABSI showed the most robust associations of all predictors with overall CVD and all CVD subcategories. ABSI demonstrated stronger correlations than BMI for all CVD outcomes (except CHF in the multivariable model). This study attempted to create classifications of ABSI and compare them to the normative classifications of BMI. In this categorical analysis, ABSI was also stronger than BMI in all logistic regression analyses for CVD outcomes, except for CHF in the multivariable model. Severe obesity (BMI ≥40 kg/m^2^) almost doubled the odds of having CVD, while being categorized in Q2, Q3, and Q4 for ABSI increased odds by double, triple, and eight-fold, respectively.

Conclusion: An ABSI parameter in the upper three quartiles increases the risk of CVD manifestations more significantly than an elevated BMI per category of overweight, obese, and severely obese, respectively. Since the categories for ABSI were created based on quartiles of a large sample size reflecting the US population, this suggests that the increased risk from an elevated ABSI is more widespread than previously understood. Thus, ABSI should be monitored more closely and managed in preventative medical care than BMI alone.

## Introduction

Recent research in cardiovascular medicine has increased understanding of modifiable risk factors for cardiovascular disease (CVD). The current over-consumption of high-calorie foods and sedentary lifestyles has contributed to the emergence of new risks factors for CVD: Obesity and type-2 diabetes mellitus [1]. Obesity is an epidemic in the United States. According to data from the National Health and Nutrition Examination Survey (NHANES) 2017-2018, 44% of men and 43% of women were classified as obese (BMI ≥30 kg/m^2^) [2]. Obesity is classified according to body mass index (BMI), calculated as weight in kilograms divided by the square of height in meters: Overweight and obese are categorized as a BMI of 25.0-29.9 kg/m^2^ and ≥30.0 kg/m^2^, respectively. Obesity is then subdivided into obesity class I (BMI 30.0-34.9 kg/m^2^), obesity class II (BMI 35.0-39.9 kg/m^2^), and obesity class III (severe/morbid obesity) (BMI ≥40 kg/m^2^) [3]. From 2003 to 2018, a period of eight NHANES surveys, the mean BMI has significantly increased between each successive survey for both men by an average of 0.18 kg/m^2^ [CI = 0.11, 0.25; p<0.0001] and women by 0.24 kg/m^2^ [CI = 0.14, 0.33; p<0.0001] per two-year NHANES cycle [2]. BMI is the most common index of adiposity used in clinical practice, and numerous studies have shown a clear relationship between BMI and comorbidities [4]. Using BMI to classify obesity has several limitations. First, the weight measurement used in the calculation does not distinguish between lean body mass and fat mass [5]. One study found that BMI ≥30 kg/m^2^ had good specificity in both men and women (95% and 99%, respectively) for ruling-out obesity but had a low sensitivity of 36% in men and 49% in women for diagnosing the “gold-standard” definition of obesity: body fat >25% in men and body fat >30% in women [6]. Secondly, BMI may not generalize due to age, sex, and ethnic differences [5]. Computed tomography (CT) and magnetic resonance imaging (MRI) are the gold standards for assessing body composition since they can accurately discriminate abdominal fatty components. However, these tests are expensive and require special equipment [6].

In the 1950s, two different types of obesity were described: gynoid obesity, the “low-risk” type, characterized by localization of fat on the lower part of the body, and android obesity, characterized by localization of fat in the upper body. This “high-risk” type, also known as central obesity, is associated with insulin resistance, atherosclerosis, type II diabetes, and CVD [1,7]. Central obesity is due to an accumulation of abdominal fat stored both subcutaneously (subcutaneous adipose tissue; SAT) and in the abdominal cavity (visceral adipose tissue; VAT). High levels of VAT rather than SAT increase diabetogenic and atherogenic metabolic risks. Factors associated with adiposity differences include sex, age, race/ethnicity, hormonal profile, smoking, nutrition, and physical activity [6].

Waist circumference (WC) has been adapted as a surrogate marker for assessing central obesity due to its ease of use, inexpensiveness, and correlation with abdominal imaging [6,8]. It is measured using a tape measure with the patient standing at the end of expiration. Sagittal abdominal diameter (SAD) measures the trunk’s thickness as the distance from the small of the back to the abdomen while the patient is in the supine position. While in this position, accumulated visceral fat maintains the height of the core in the sagittal direction. Subcutaneous fat reduces the height of the abdomen due to gravity [9,10].

Measuring WC or SAD alone does not separate the impact of body shape from body size (height, weight, and BMI). Using data from the NHANES 1999-2004 to construct the model, a body shape index (ABSI) was developed based on WC (body shape) adjusting for height and weight: ABSI = WC / (BMI^2/3^ • Height^1/2^) [11]. This study used mortality outcomes from the National Death Index, representing two to eight years of follow-up, to quantify mortality risk. A higher ABSI has been shown to increase mortality hazard and an increase in the population. ABSI by one standard deviation increases the risk of death by 33% [95% CI = 20%-48%] [11].

## Materials and methods

The present study examined the correlation of ABSI with prior manifestations of overall CVD and subcategories of CVD, including angina pectoris (AP), coronary artery disease (CAD), congestive heart failure (CHF), myocardial infarction (MI), and stroke. For comparison, the same outcome variables are correlated with BMI and other anthropometric measures of central obesity, WC, and SAD. This study aims to determine the utility of ABSI as a predictor of CVD compared to other currently used measures in clinical practice.

Description of data

Public-use data from the baseline interview and health examination from the National NHANES 2013-2014 was used. NHANES 2011-2014 was approved by the National Center for Health Statistics Ethics Review Board under Protocol #2011-17 [12].

NHANES is designed to collect a nationally representative sample of the resident, civilian, non-institutionalized U.S. population. NHANES has been designed to oversample Hispanic persons, non-Hispanic black persons, low-income white persons (≤130% of federal poverty level), and adults over the age of 80 to better understand the health of these sub-populations. The primary change in the NHANES 2011-2014 design was an addition of an oversample of the Asian sub-population [12].

The data set from which the current analysis was done required the survey participants to complete a series of questionnaires administered through a computer-assisted personal interviewing system, followed by a standardized examination in a mobile examination center. The interview consisted of questions on a spectrum of health-related risks, attitudes, and behaviors. Demographic variables included age (due to privacy concerns, those 80 years and older were coded as 80 years), gender, and ethnicity (Mexican American, other Hispanic, non-Hispanic white, non-Hispanic black, non-Hispanic Asian, and other race/multi-racial) [13].

Trained health technicians and recorders collected anthropomorphic measures during the health examination. Weight was measured in kilograms (kg) using a digital weight scale. Height was measured in centimeters (in all participants two years and older who can stand unassisted) using a wall-mounted stadiometer. WC was measured in centimeters at the uppermost lateral borders of the ilium using a tape measure. The participant was placed in the dorsal recumbent position to obtain the SAD that was measured in centimeters using an abdominal caliper at the iliac level during exhalation. This measure was repeated one to three times (between two to four trials per participant) [14]. BMI was calculated using the equation BMI = weight (kg) / Height (m)^2^. ABSI was calculated using the equation ABSI = WC (m) / [BMI^2/3^ (kg/m^2^) • Height^1/2^ (m)] [11].

Statistical analysis

All non-pregnant adults (18-80 years) were included for analysis with a sample size of 5,924 participants. Analysis was done using R Studio, version 0.99.903 (RStudio, Inc. Boston, MA). Values for BMI, WC, and SAD were ascertained from the NHANES data. ABSI was calculated using ABSI = WC / (BMI^2/3^ • Height^1/2^). These independent predictors were treated as continuous variables. The primary outcome, any past manifestation of CVD, was a binary variable coded as “yes” if the individual responded “yes” to any of the sub-categories. The secondary outcome variables (sub-categories of CVD) were self-reported binary responses from the health questionnaire portion of the NHANES were as follows:

· For CHF - Were you ever told that you had congestive heart failure (MCQ160b)?

· For CAD - Were you ever told that you had coronary heart disease (MCQ160c)?

· For AP - Were you ever told that you had angina/angina pectoris (MCQ160d)?

· For MI - Were you ever told that you had a heart attack (MCQ160e)?

· For stroke - Were you ever told that you had a stroke (MCQ160f)?

Covariates of interest included demographic variables such as age, gender, and ethnicity. Other confounders included the following binary responses from the health questionnaire:

· For high blood pressure - Has a doctor ever told you that you have high blood pressure (BPQ020)?

· For high cholesterol - Has a doctor ever told you that you have a high cholesterol level (BPQ080)?

· For diabetes - Has a doctor ever told you that you have diabetes (DIQ010)?

· For smoking - Have you smoked at least 100 cigarettes in your life (SMQ020)?

All “no response” answers were re-coded as “NA” and treated as such in the analysis. Individuals who answered “borderline” to diabetes (DIQ010) were re-coded as “yes.” We used an a priori significance level of 0.05.

The correlation between body size and shape measures was tested using Pearson’s correlation. For the logistic regression of CVDs and ABSI, an unadjusted model and a multivariable model that initially included all possible confounders (age, gender, ethnicity, high blood pressure, diabetes, and smoking) were used. Then potential confounders that were non-significant were eliminated in a stepwise fashion to find the final multivariable model. This process was then repeated for BMI, WC, and SAD. 

In a secondary analysis, ABSI was reclassified into categorical values based on the quartile ranges for the NHANES dataset: Quartile 1 (Q1), went from an ABSI of 0.064 to 0.078; Quartile 2 (Q2), >0.078 to 0.082; Quartile 3 (Q3), >0.082 to 0.085; and Quartile 4 (Q4), >0.085 to 0.108. Logistic regressions were again run for overall CVD and all sub-categories of CVD manifestations. Chi-square tests were used to test for significance.

For comparison, logistic regressions were done with BMI categorized as normal (<25 kg/m^2^), overweight (25-29.9 kg/m^2^), obese (30-39.9 kg/m^2^), and severely obese (≥40 kg/m^2^). WC and SAD do not have established normative classifications as BMI does. BMI was tested with overall CVD and all subcategories as outcome measures. Chi-square tests followed this analysis to assess for significance

## Results

**Figure 1:**
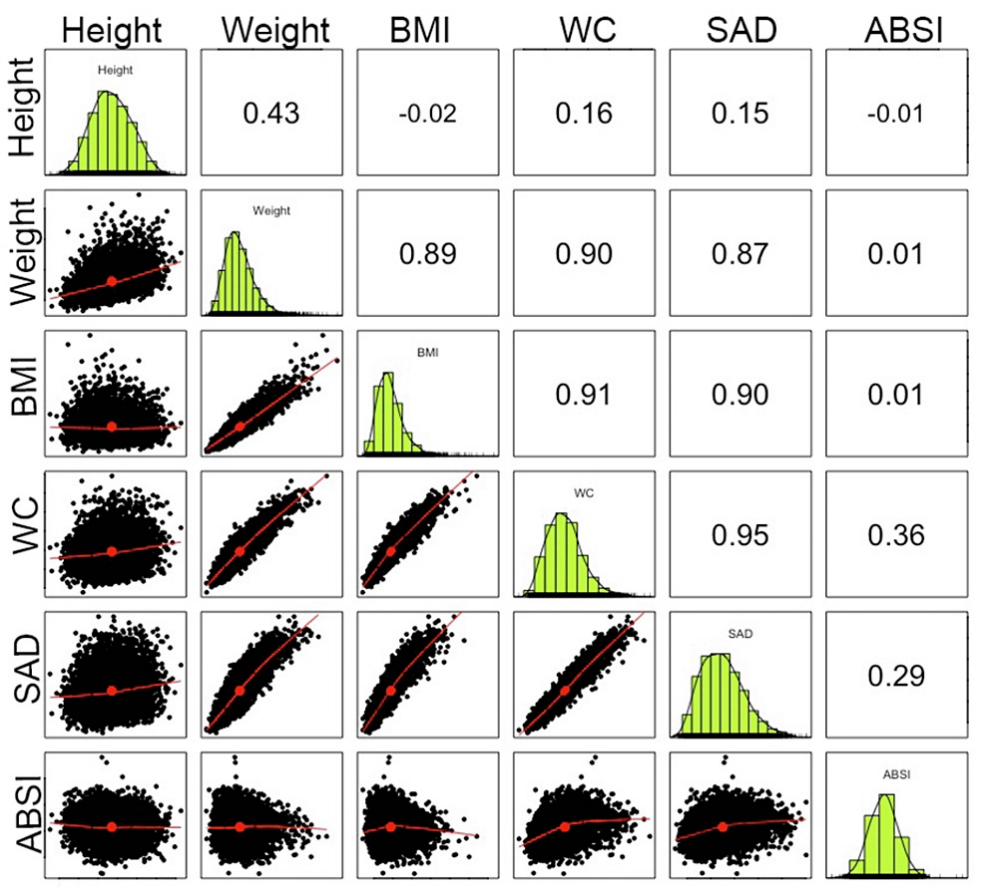
Correlation of Anthropometric Measures Between Body Size and Shape Data Matrix: diagonal axis shows histograms of data distribution for each anthropometric measure. Numeric entries are Pearson correlations between each anthropometric measure; bottom row shows Scatter Plot Matrix (SPLOM) for ABSI compared with the other measures; ABSI shows a moderate correlation with WC (r = 0.36), and a weak correlation (r <0.3) with height, weight, BMI, and SAD. SPLOM for Weight x BMI (r = 0.89), Weight x WC (r = 0.90), Weight x SAD (r = 0.87), BMI x WC (r = 0.91), BMI x SAD (r = 0.90), WC x SAD (r = 0.95) all demonstrate a strong positive linear relationship. BMI = Body Mass Index; WC = Waist Circumference; SAD = Sagittal Abdominal Diameter; ABSI = A Body Shape Index. Image created by the authors.

Characteristics of the sample population are shown in Table 1. Figure 1 illustrates the correlation between body size and shape indicators. Weight, BMI, WC, and SAD have a strong linear correlation (r ≈ 0.9). ABSI shows a weak correlation with height, weight, BMI, and SAD (r<0.3) and only a moderate correlation with WC (r = 0.36) which is the numerator of the ABSI equation. Its low correlation demonstrates that ABSI is a distinct measure from the other anthropometric predictors.

**Table 1:** Demographics of the Sample Population BMI = Body Mass Index; WC = Waist Circumference; SAD = Sagittal Abdominal Diameter; ABSI = A Body Shape Index

	Median	Mean	Std. Dev.
Age (years)	47.00	47.41	18.43
Height (cm)	166.70	167.09	10.16
Weight (kg)	77.70	81.00	22.17
BMI (kg/m^2^)	27.70	28.92	7.19
WC (cm)	97.00	98.45	16.82
SAD (cm)	22.10	22.52	4.53
ABSI	0.0815	0.0815	0.0049

Table 2 shows the proportion of the sample population by categorical characteristics and possible risk factors for CVD: diabetes (9.4%), high blood pressure (32.6%), high cholesterol (32.6%), and smoking (41.7%). Table 3 summarizes the proportion of responses for total CVD events and subcategories of CVD. Approximately 10% of the sample population reported at least one prior manifestation of CVD. The most common cardiovascular event was MI (4.0%), while AP was the least common (2.3%).

**Table 2:** Categorical Characteristics of the Sample Population Diabetes = Participant was told by a doctor that they have diabetes; High Blood Pressure = Participant was told by a doctor that they have high blood pressure; High cholesterol = Participant was told by a doctor that they have high cholesterol; Smoking = Smoked at least 100 cigarettes in life.

	Percent (n)
Gender	
Female	52% (3101)
Male	48% (2823)
Race	
Non-Hispanic White	42% (2491)
Non-Hispanic Black	20.6% (1223)
Mexican American	14% (830)
Other Race/Multiracial	11.4% (675)
Other Hispanic	8.8% (523)
Non-Hispanic Asian	3.1% (182)
Diabetes	
No	90.6% (5363)
Yes	9.4% (557)
High Blood Pressure	
No	64.5% (3815)
Yes	35.5% (2104)
High Cholesterol	
No	67.4% (2737)
Yes	32.6% (1324)
Smoking	
No	58.3% (2249)
Yes	41.7% (1608)

**Table 3:** History of Cardiovascular Disease (CVD) Manifestations in the Sample Population Proportion of participant responses to being told they had [cardiovascular disease] by a doctor.

	Percent (n)
Any past manifestation of CVD	
No	90.3% (5350)
Yes	9.7% (574)
Angina Pectoris	
No	97.7% (5451)
Yes	2.3% (131)
Coronary Artery Disease	
No	96.0% (5348)
Yes	4.0% (224)
Congestive Heart Failure	
No	96.8% (5404)
Yes	3.2% (177)
Myocardial Infarction	
No	96.0% (5363)
Yes	4.0% (222)
Stroke	
No	96.5% (5389)
Yes	3.5% (194)

Using logistic regression, the correlation between each predictor (ABSI, BMI, WC, SAD), and cardiovascular events was evaluated using two models. One was a univariable model with only the predictor and outcome variable and the other was a multivariable model adjusted for confounders that were found to be significant. All the anthropometric predictors were significantly associated with CVD events (p<0.001). The odds ratio (OR) for ABSI was 1.17 [CI 1.15-1.20], for BMI was 1.03 [1.02-1.04], for WC was 1.03 [1.02-1.03], and for SAD was 1.11 [1.08-1.13]. In the stratified analysis, ABSI, WC, and SAD remained significant predictors for all subcategories of CVD (p<0.001). BMI remained significant only for AP (p<0.05), CHF (p<0.001), and MI (p<0.05).

For the multivariable logistic regression, the final models for each predictor included gender, age, and high blood pressure. High cholesterol was included in the models for BMI and WC. Interactions were initially included but found to be non-significant and therefore removed. The results of the logistic regression for all the anthropometric predictors were significant for CVD manifestations overall (p<0.001): ABSI OR = 1.05 [CI 1.02-1.07], BMI OR = 1.04 [CI 1.02-1.05], WC OR = 1.02 [CI 1.01-1.03], and SAD OR = 1.06 [CI 1.03-1.08].

In the analysis of the subcategories, all the predictors were significantly associated with CAD (BMI and SAD p<0.05; ABSI and WC p<0.01) and MI (BMI p<0.05; SAD p<0.01; ABSI and WC p<0.001). For AP, ABSI (p<0.01), WC (P<0.01), and SAD (p<0.05) were significant predictors. For CHF, BMI (p<0.001), WC (p<0.001), and SAD (p<0.001) were significant. Lastly, only ABSI (p<0.05) was a significant predictor in the multivariable model for stroke. Table 4 summarizes all anthropometric predictors and CVD outcomes for both the univariable and multivariable models.

**Table 4:** Odds Ratios for Cardiovascular Disease Outcomes ^a^ Multivariable model for ABSI and SAD includes age, gender, high blood pressure; ^b^ Multivariable model for BMI and WC includes age, gender, high blood pressure, and high cholesterol; Odds Ratio OR [95% CI]; Significance p-value:  <0.05* <0.01** <0.001*** BMI = Body Mass Index; WC = Waist Circumference; SAD = Sagittal Abdominal Diameter; ABSI = A Body Shape Index; CVD = Cardiovascular Disease; AP = Angina Pectoris; CHF = Congestive Heart Failure.

		CVD	AP	CAD	CHF	MI	Stroke
ABSI	Univariable	1.17*** [1.15-1.20]	1.16*** [1.11-1.20]	1.18*** [1.14-1.21]	1.13*** [1.09-1.17]	1.19*** [1.15-1.23]	1.14*** [1.15-1.23]
	Multivariable ^a^	1.05 *** [1.02-1.07]	1.06** [1.01-1.11]	1.05** [1.01-1.09]	1.03 [0.99-1.07]	1.09*** [1.05-1.13]	1.04* [1.00-1.08]
BMI	Univariable	1.03*** [1.02-1.04]	1.02* [1.00-1.05]	1.02 [1.00-1.04]	1.06*** [1.04-1.08]	1.02* [1.00-1.04]	1.00 [0.98-1.02]
	Multivariable ^b^	1.04*** [1.02-1.05]	1.03 [1.00-1.06]	1.03* [1.00-1.05]	1.07*** [1.05-1.10]	1.03** [1.01-1.06]	1.00 [0.97-1.03]
WC	Univariable	1.03*** [1.02-1.03]	1.03*** [1.02-1.04]	1.02*** [1.02-1.03]	1.03*** [1.02-1.04]	1.03*** [1.02-1.03]	1.02*** [1.01-1.02]
	Multivariable ^b^	1.02*** [1.01-1.03]	1.02** [1.01-1.03]	1.02** [1.01-1.03]	1.02*** [1.02-1.05]	1.02*** [1.01-1.03]	1.01 [1.00-1.02]
SAD	Univariable	1.11*** [1.08-1.13]	1.10*** [1.05-1.14]	1.09*** [1.06-1.13]	1.14*** [1.10-1.18]	1.10*** [1.07-1.14]	1.07*** [1.03-1.10]
	Multivariable ^a^	1.06*** [1.03-1.08]	1.06* [1.01-1.10]	1.04* [1.01-1.08]	1.10*** [1.06-1.15]	1.06** [1.07-1.14]	1.02 [0.98-1.07]

Table 5 shows the z-scores associated with the univariable logistic regression analyses and each of the predictors (ABSI, BMI, WC, and SAD) and each of the CVD outcomes. As stated before, a significant correlation was found in the univariable analysis with almost all the predictors and outcomes (z-score >1.96). BMI as a predictor for CAD (z-score = 1.94) and stroke (z-score = 0.33). While significant for the other outcomes, BMI had the lowest compared to the other predictors: CVD (z-score = 4.75), AP (z-score = 2.16), CHF (z-score = 6.65), and MI (z-score = 2.50). ABSI had the highest z-score for each of the outcome variables: overall CVD (z-score = 15.6), AP (z-score = 7.74), CAD (z-score = 10.7), CHF (z-score = 7.28), MI (z-score = 11.3), and stroke (z-score = 8.22). The z-scores of WC and SAD were similar for all univariable analyses.

**Table 5:** Comparison of Z-Statistics from Univariable Logistic Regression Analyses BMI = Body Mass Index; WC = Waist Circumference; SAD = Sagittal Abdominal Diameter; ABSI = A Body Shape Index; CVD = Cardiovascular Disease; AP = Angina Pectoris; CHF = Congestive Heart Failure; Significance p-value <0.05* <0.01** <0.001***

	CVD	AP	CAD	CHF	MI	Stroke
ABSI	15.6***	7.74***	10.7***	7.28***	11.3***	8.22***
BMI	4.75***	2.16*	1.94	6.65***	2.50*	0.33
WC	9.71***	5.15***	5.95***	7.27***	6.65***	3.42***
SAD	10.1***	4.72***	5.80***	7.30***	6.38***	3.62***

The z-scores from the multivariable model are shown in Table 6. Compared to the other predictors, WC had the highest z-score for CVD (z-score = 4.67), AP (z-score = 2.74), and CAD (z-score = 2.70). BMI had the highest z-score for CHF (z-score = 6.15). ABSI had the highest z-score for MI (z-score = 4.60) and stroke (z-score = 1.98).

**Table 6:** Comparison of Z-Statistics from Multivariable Logistic Regression Analyses ^a^ Multivariable model for ABSI and SAD includes age, gender, high blood pressure; ^b^ Multivariable model for BMI and WC includes age, gender, high blood pressure, and high cholesterol; Significance p-value <0.05* <0.01** <0.001*** BMI = Body Mass Index; WC = Waist Circumference; SAD = Sagittal Abdominal Diameter; ABSI = A Body Shape Index; CVD = Cardiovascular Disease; AP = Angina Pectoris; CHF = Congestive Heart Failure.

	CVD	AP	CAD	CHF	MI	Stroke
ABSI ^a^	3.8***	2.65**	2.66**	1.28	4.6***	1.98*
BMI ^b^	4.3***	1.84	2.23*	6.15***	2.83**	0.33
WC^ b^	4.67***	2.74**	2.7**	5.16***	3.72***	1.24
SAD ^a^	4.57***	2.41*	2.35*	4.9***	3.14**	1.11

Table 7 shows the results of the categorical analysis of ABSI quartiles and BMI according to their standard categories. The multivariable models in the categorical outcome analysis included the same confounders as those in the continuous outcome analysis: ABSI had age, gender, and history of high blood pressure. The BMI multivariable model included age, gender, history of high blood pressure, and high cholesterol. ABSI by category was a significant predictor (p<0.001) for all CVD outcomes in the univariable model. Having an ABSI parameter in Q2 or Q3 doubled and tripled the risk for overall CVD, respectively. At the same time, an ABSI parameter in Q4 increased risk by eight-fold. BMI by category was only a significant predictor (p<0.001) for CVD overall and MI, and unlike BMI (continuous), it was no longer significant for AP and MI. The categories of overweight (BMI >25-30 kg/m^2^) and obese (BMI >30-40 kg/m^2^) both increased risk of CVD by 49%, while classification as severely obese (BMI >40 kg/m^2^) nearly doubles risk compared to a normal BMI (<25 kg/m^2^).

**Table 7:** Odds of Cardiovascular Disease Manifestations by Categorical Analysis of ABSI and BMI ABSI – Q1 (0.064-0.078), Q2 (>0.078-0.082), Q3 (>0.082-0.085), Q4 (>0.085-0.108); BMI – Normal (<25 kg/m^2^), Overweight (25-29.9 kg/m^2^), Obese (30-39.9 kg/m^2^), Severe Obese (≥40 kg/m^2^); ^a^ Multivariable model for ABSI includes age, gender, high blood pressure; ^b^ Multivariable model for BMI includes age, gender, high blood pressure, and high cholesterol; Significance p-value <0.05* <0.01** <0.001*** ABSI = A Body Shape Index, BMI = Body Mass Index; CVD = Cardiovascular Disease; AP = Angina Pectoris; CHF = Congestive Heart Failure.

		CVD		AP		CAD		CHF		MI		Stroke	
		Univariable	Multivariable	Univariable	Multivariable	Univariable	Multivariable	Univariable	Multivariable	Univariable	Multivariable	Univariable	Multivariable
ABSI ^a^	p-value	<0.001***	<0.001***	<0.001***	<0.01**	<0.001***	<0.05*	<0.001***	0.12	<0.001***	<0.01**	<0.001*	0.091
	Q1	1.00	1.00	1.00	1.00	1.00	1.00	1.00	1.00	1.00	1.00	1.00	1.00
	Q2	2.04	1.13	2.32	1.55	1.38	0.77	3.23	2.07	2.26	1.40	1.58	0.98
	Q3	3.01	1.16	2.84	1.42	2.55	0.94	3.13	1.46	4.31	1.92	1.69	0.77
	Q4	8.06	1.73	9.17	2.88	7.33	1.45	7.34	1.99	10.4	2.74	4.92	1.32
BMI ^b^	p-value	<0.001***	<0.01**	0.15	0.498	0.28	0.172	<0.001***	<0.001***	0.14	0.13	0.56	0.68
	Normal	1.00	1.00	1.00	1.00	1.00	1.00	1.00	1.00	1.00	1.00	1.00	1.00
	Overweight	1.49	1.24	1.44	0.92	1.23	1.02	0.94	0.74	1.28	0.98	1.26	1.16
	Obese	1.49	1.33	1.48	1.11	1.36	1.42	1.65	1.42	1.37	1.19	1.10	0.87
	Severe Obese	1.97	2.26	2.02	1.70	1.52	1.81	3.09	3.20	1.74	2.06	0.88	1.03

In the multivariable model, ABSI by category remained significant for overall CVD (p<0.001), AP (p<0.01), CAD (p<0.05), and MI (p<0.01). ABSI in Q2, Q3, and Q4 for overall CVD increased risk by 13%, 16%, and 73% compared to an ABSI parameter in Q1. The risk for AP and MI also follows a positive trend with increasing quartiles of ABSI. However, being in Q2 (OR = 0.77) or Q3 (OR = 0.94) seems to have a protective effect on CAD, while CAD’s risk increases by 45% in Q4. BMI by category continued to be a significant predictor for both CVD (p<0.01) and CHF (p<0.001). The risk of overall CVD was increased by 24% for overweight, 33% for obese, and 126% for severely obese. The risk of CHF decreased by 26% for overweight but increased by 42% for obese and 220% for severely obese categories.

## Discussion

Over the past decades, the percentage of overweight subjects in the US population has steadily increased [15,16]. To classify overweight and obesity, several anthropometric measures have been studied as a surrogate measure for diagnosing obesity and estimating body fat distribution. Currently, the BMI and WC are recommended by the Centers for Disease Control and Prevention and World Health Organization guidelines. However, an important limitation to BMI is the lack of distinction between lean mass and adipose tissue, while it is not clear if WC depends on body shape [17,18]. These limitations have led scientists to pursue a better body fat index considering lean mass and body fat distribution. ABSI is positively associated with fat mass and negatively associated with fat-free mass, thus showing potential as an improved surrogate measure compared to BMI [19]. Additionally, there is a positive association between ABSI and VAT in patients with type 2 diabetes [20].

The ABSI was created to estimate body shape health as a quantitative measure independent of BMI, WC, and height [11]. In the present sample population, ABSI did not show an association with BMI, SAD, height, and weight, while BMI, WC, SAD, and weight were strongly associated with each other (r ≈0.90-0.95). In previous studies, ABSI predicted premature mortality better than BMI and WC. In age- and sex-adjusted models, BMI was related to CVD mortality. Still, the associations followed a “U” or “J-shaped” curve; thus, BMI did not show statistical significance with all-cause mortality until BMI >30 kg/m^2^ in all patients, or >25 kg/m^2^ in healthy non-smoking patients [21].

ABSI shows a linear relationship to the risk of developing CVD in men and women without a threshold being detectable. Additionally, the magnitude of the hazards ratio for CVD conferred by ABSI was higher than those granted by other anthropometric measures. Although it could not improve predictability, it provided more information than traditional anthropometric measures [22]. It was previously found that ABSI was strongly associated with metabolic syndrome and VAT thickness. The everyday use of ABSI and BMI is a strong predictor of low HDL (high-density lipoprotein) cholesterol, high triglycerides, and elevated fasting glucose compared to BMI alone [23].

The present results suggest that uncorrected, ABSI does have much more robust associations with prior manifestations of CVD (z-score = 15.6) and each of the sub-categories (z-score range = 7.28-11.3). Thus, an increase in WC by one cm increases the odds of CVD by 3%, and an increase in SAD by one cm increases the odds by 11%. The interpretation of the odds for BMI and ABSI is less intuitive.

In the adjusted model, WC had the highest z-score for any CVD, AP, and CAD, and BMI had the highest z-score for CHF. ABSI continued to have the highest z-score for MI and stroke, while all other predictors lost statistical significance for stroke. According to previous literature, ABSI has been shown to underperform compared to BMI and WC in predicting chronic diseases but has outperformed in predicting all-cause mortality [24].

CVD is multifactorial with several potential confounders, including age, gender, health behaviors (smoking, physical activities, diet), and health factors (high blood pressure, high cholesterol, and high glucose). A meta-analysis of 38 studies showed a strong correlation of ABSI with hypertension, type 2 diabetes, CVD, and all-cause mortality. A standard deviation increase in ABSI increased the odds of hypertension by 13% (OR = 1.13 [1.04-1.22]), diabetes by 35%, CVD by 21% (HR = 1.21 [1.10, 1.32]), and all-cause mortality by 55% [24]. ABSI has been shown to predict CVD with accuracy like laboratory-based predictors (lipid profiles, such as total cholesterol and HDL cholesterol) [25]. In the present multivariable analysis, it was found that gender, age, and a previous diagnosis of high blood pressure were significant covariates for all anthropometric predictors. High cholesterol was significant in the multivariable models for BMI, WC, and ABSI. However, it was excluded from the final model for ABSI because ABSI is predictive of hyperlipidemia, thus being a likely mediator.

In the multivariable model, WC had the highest z-score for CVD (z-score = 4.67), AP (z-score = 2.74), and CAD (z-score = 2.70). BMI had the highest z-score for CHF (z-score = 6.15). ABSI had the highest z-score for MI (z-score = 4.60) and stroke (z-score = 1.98). This suggests that WC has the highest specificity when accounting for gender, age, history of hypertension, and hyperlipidemia. This may be useful for patients whose health history is known and being monitored.

While these logistic regressions with the predictors as continuous variables are useful statistically for predicting risks, they have less utility for clinical usage. As stated earlier, ABSI, WC, and SAD do not have established normative categories. In this study, an attempt was made to create normative classifications based on the quartiles of a large sample population. For that purpose, ABSI was classified in quartiles as Q1, ranging from 0.064 to 0.078; Q2, >0.078 to 0.082; Q3, >0.082 to 0.085; and Q4, >0.085 to 0.108. An ABSI value in Q2 and Q3 more than doubled and tripled the risk of having a CVD manifestation, respectively. At the same time, being in Q4 increased risk more than eight-fold. BMI, which has normative categories, has a much lower risk increase with each category than ABSI: 49% for overweight and obese and 97% for severely obese. However, while stratified by sub-categories of CVD, BMI remained significant for CVD (p<0.001), CAD (p<0.001), and CHF (p<0.001). ABSI remained significant (p<0.001) for all sub-categories of CVD manifestations except for CHF.

These results suggest the utility of ABSI as a predictor in clinical practice. Unlike other ABSI studies that focus on mortality risk, the present study underestimates the mortality risk of these CVD outcomes since the study participants had these manifestations before data collection. Additionally, further evaluation of ABSI as a modifiable risk factor would include decreasing WC (the numerator) while increasing BMI, suggesting that an increase in peripheral lean mass protects against CVD manifestations. Because the risks for ABSI are linear, finding the ideal range for ABSI versus the absolute minimum is needed, especially in the US population. One study found a cut-off value of an ABSI parameter of 0.078 for low risk of coronary heart disease in Chinese males. This study found ABSI to be the best index for predicting coronary heart disease risk in males of this population but was less suitable compared to waist-to-height ratio and body roundness index in Chinese females. Nonetheless, ABSI still outperformed BMI in both genders [26].

Limitations of the study

The history of CVD was self-reported. Although the questionnaire implied that physicians diagnosed the outcome, responses might be underreported or over-reported. We did not perform an area under the receiver operating characteristic (AROC) analysis to evaluate the predictors’ discriminative power and improve the accuracy of the results. Additionally, the study’s cross-sectional design has limited capacity to demonstrate the predictive validity of anthropometric measures. 

## Conclusions

The purpose of this study was to determine the utility of ABSI as a predictor of CVD compared to other currently used measures in clinical practice. In this study, comparisons using quartiles of a large sample size (n >5,500) suggest that an increased risk from an elevated ABSI is common and essentially overlooked. Additionally, the increase in risk per category of ABSI is much greater in magnitude than an increase per category of BMI. This insinuates that an elevated ABSI is a major risk factor that has more drastic chances of consequences. Therefore, this parameter should be monitored more closely and managed in preventative medical care. Further, the current findings suggest that ABSI is an improved measure for diagnosing obesity and estimating body fat distribution. This is significant because obesity has been shown to be associated with several co-morbidities contributing to metabolic disease and continues to be an epidemic in the developing world. Using BMI to classify obesity has several limitations, most importantly it cannot discern between lean/fat mass or accounting for body fat distribution. Central obesity, characterized by an accumulation of subcutaneous and visceral fat in the trunk, is a “high-risk” factor for CVD that can be discriminated by ABSI.
